# The Way They Died Then

**Published:** 1890-03-22

**Authors:** 


### The Way They Died Then.

Nearly a hundred years ago—to be accurate, in August, 1795—Mrs. Davenport died at Leicester. So far as we know, Mrs. Davenport was wholly unknown to fame, and the utmost we can contribute to her biography, from the materials at our command, is this: That on February 7th, 1795, she felt so ill that she called in Dr. John Hunt to attend her, and that he prescribed and sent her "a draught," for which he charged one shilling. During February and March Mrs. Davenport consumed medicine at a steady rate, which makes us envy her constancy. Every alternate day saw a new bottle brought in for her use, save when a box of pills, priced at 2*s.* 6*d.*, arrived by way of a change. If she was a frugal woman, which we incline to believe from her subsequent conduct, she would rejoice when the doctor sent only the modest shilling draught. Other doses—juleps and decoctions—were priced at three shillings each, and do not seem to have lasted longer than the regulation forty-eight hours. Perhaps, however, they were less nauseous than the cheaper article. The nature of the drugs thus administered remains unknown; one or two dates have appended to the note "bark decoction"; but for the rest a judicious vagueness overhangs the doctor's bill, which forms the sole remaining record of Mrs. Davenport's last days.

Some things there are that raise a surmise in the imaginative mind. The end of February and the first week of March saw Mrs. Davenport at her worst. At least, so we guess—and it is in this fashion that much history is written—from the fact that although on the 24th of February she had received a bark decoction and a box of pills, the 25th saw a new decoction, and the 26th is marked by the administration of two draughts. Two draughts—two varieties of black unpalatableness. And for the next day, and the next again, is set down a "ditto," which arouses in all who have tongues and stomachs sympathy for that long dead suffering woman. The 1st of March was a memorable day in the record of Mrs. Davenport's illness. She must have been in a very bad way, for the list of her medicaments for that 1st of March runs thus: "Two draughts, 2s.; a decoction, 3s.; two draughts, 2s." Five different doses in one day! Poor Mrs. Davenport!

Either she rebelled against the treatment or she got better. At least she determined to see what country air would do for her and fled from Leicester to Cotes, a village near Loughborough. This we infer from the fact that suddenly the draughts and decoctions cease, and after an interval of a fortnight we come on the item, "Journey to Cotes, 2s. 6d." Now Cotes is about nine miles from Leicester. What doctor of these days would drive nine miles and back for half-a-crown? For this is all he got; it is not merely the payment of travelling expenses. Dr. John Hunt never made any charge for his visits; the advice he gave was gratuitous; he made his money out of the draughts and decoctions. No wonder he "threw in" the medicine with such a vigorous hand. Dear patients, a word with you. Next time you feel inclined to complain of the fee your doctor asks for advising you to wear flannel, or to sleep with your window open o' nights, just ask yourself if it is not better to spend your money for the receipt of a little simple sanitary truth than to force the poor medicine man, on pain of starvation, to inflict on you the perhaps harmless, but certainly unappetising, draught or decoction, with perhaps a bleeding—price one shilling—thrown in by way of variety.

After all Dr. Hunt did not make very much out of his patient. For although his attendance lasted till at least the 6th of June, when "a journey" is set down, the total account reaches only the modest sum of £4 12s. Medicine is not even now the best paid of the so-called "liberal professions"; but still things are not so bad as in 1795, when five months' attendance was recompensed by something under five pounds.

With regard to death, and the expenses thereon depending, one might say with Macbeth, "If 'twere done when it were done, then 'twere well." But it isn't. After the doctor has departed comes what Wendell Holmes calls the post-medical profession, whose members strive to atone for the hardships of life by making death a luxury. In these latter days, indeed, there has arisen a mean-tempered society, entitled the Funeral Reform Association, which tries to make people believe that they can, with decency, be buried as simply as they lived. They had not got to reforms in 1795, and Mrs. Davenport was not haunted in her last moments by the dread of a wicker coffin—not to hint at cremation—or a grave some inches less than six feet deep.

Do the dying, indeed, think of these things? In the face of the great change before them, when they are about to go forth into the dark, whether with or without hope of the light beyond, are their souls indeed vexed by questions of funeral baked meats, of hat-bands and palls? Is it not rather the survivors, half dazed with loss, heartbroken at the thought of cold words spoken, services withheld, who thus say, in such poor form as mortal effort can—"Dear, we loved you; all we have is yours." O that grief should become food for cupidity!

Yet there is something to be said for the undertaker. His is a necessary, though ungrateful task. He is little more popular in society than the hangman, though perhaps more necessary. What wonder, then, that he should take revenge for the slights that are cast upon his calling. Then it must be admitted that life treats him ill. "You see, sir, it's our only chance," was the plea offered by one of the fraternity to a lawyer who, bold man, disputed the charges for a deceased client's funeral. Perhaps the excuse is a sufficiently good one. At least, Mr. Ellis Brewin, of Leicester, acted on it to the extent of making Mrs. Davenport's funeral nearly ten times as expensive as her last illness had been. It is easy to make up a bill when one can charge over five pounds for hat-bands. There were seventeen of these hat-bands, each two yards long. Is there a man now living who would go forth into the streets with two yards of black silk streaming from his hat? The Funeral Reform Association has given a quietus to aggressive mourning of that description. We may still pay a guinea for the temporary use of a "best double trim'd velvet pall," but the streaming hat-bands and the waving scarves—two of these cost £1 15s. 9d.—are things of the past. Even the bestowal of gloves, black, white, and grey, on mourners has fallen into comparative desuetude. At Mrs. Davenport's funeral these cost £4 12s. 6d. We refrain from quoting further details of this account, lest some sad undertaker should fall a-weeping for these good old times, when one could charge for the funeral equipments of one middle-class lady—not including, be it noted, the price of the coffin, or of conveyance to the grave—no less than £40 16s. 7½d. But comparing this sum with the doctor's modest £4 12s., one cannot help wondering whether the grave-faced physician, or the yet graver-faced man who ultimately succeeds him, deserves to be ranked as belonging to a liberal profession. If by "liberal" we allude to the treatment given by the public, the answer is clear.

